# Effects of adenosine and regadenoson on hemodynamics measured using cardiovascular magnetic resonance imaging

**DOI:** 10.1186/s12968-017-0409-8

**Published:** 2017-12-04

**Authors:** Dustin M. Thomas, Matthew R. Minor, James K. Aden, Christopher J. Lisanti, Kevin E. Steel

**Affiliations:** 1 Cardiology Division, San Antonio Military Medical Center, San Antonio, TX USA; 2 Department of Radiology, San Antonio Military Medical Center, San Antonio, TX USA; 3 Graduate Medical Education, San Antonio Military Medical Center, San Antonio, TX USA; 4 Deputy Chief Scientist, 59 MDW/ST 2200 Bergquist Drive, JBSA-Lackland, Texas, 78236 USA

**Keywords:** Stress CMR, Adenosine, Regadenoson, Lexiscan®, Ejection fraction, Ventricular volumes

## Abstract

**Background:**

Adenosine or regadenoson vasodilator stress cardiovascular magnetic resonance (CMR) is an effective non-invasive strategy for evaluating symptomatic coronary artery disease. Vasodilator injection typically precedes ventricular functional sequences to efficiently reduce overall scanning times, though the effects of vasodilators on CMR-derived ventricular volumes and function are unknown.

**Methods:**

We prospectively enrolled 25 healthy subjects to undergo consecutive adenosine and regadenoson administration. Short axis CINE datasets were obtained on a 1.5 T scanner following adenosine (140mcg/kg/min IV for 6 min) and regadenoson (0.4 mg IV over 10 s) at baseline, immediately following administration, at 5 min intervals up to 15 min. Hemodynamic response, bi-ventricular volumes and ejection fractions were determined at each time point.

**Results:**

Peak heart rate was observed early following administration of both adenosine and regadenoson. Heart rate returned to baseline by 10 min post-adenosine while remaining elevated at 15 min post-regadenoson (*p* = 0.0015). Left ventricular (LV) ejection fraction (LVEF) increased immediately following both vasodilators (*p* < 0.0001 for both) and returned to baseline following adenosine by 10 min (*p* = 0.8397). Conversely, LVEF following regadenoson remained increased at 10 min (*p* = 0.003) and 15 min (p = 0.0015) with a mean LVEF increase at 15 min of 4.2 ± 1.3%. Regadenoson resulted in a similar magnitude reduction in both LV end-diastolic volume index (LVEDVi) and LV end-systolic volume index (LVESVi) at 15 min whereas LVESVi resolved at 15 min following adenosine and LVEDVi remained below baseline values (*p* = 0.52).

**Conclusions:**

Regadenoson and adenosine have significant and prolonged impact on ventricular volumes and LVEF. In patients undergoing vasodilator stress CMR where ventricular volumes and LVEF are critical components to patient care, ventricular functional sequences should be performed prior to vasodilator use or consider the use of aminophylline in the setting of regadenoson. Additionally, heart rate resolution itself is not an effective surrogate for return of ventricular volumes and LVEF to baseline.

## Background

Adenosine, regadenoson, and dipyridamole are common vasodilator stress perfusion agents used while performing cardiovascular magnetic resonance (CMR) stress perfusion imaging [1, 2]. When comparing stress vasodilator agents, adenosine and regadenoson were found to induce similar increases in myocardial blood flow and demonstrated superior hyperemic responses to dipyridamole in healthy subjects [3]. Additionally, adenosine was shown to be superior to dipyridamole with respect to the sensitivity and specificity for detection of obstructive coronary artery disease (CAD) (stenosis > = 50%) [4]. The selection of which stress agent to use is based on cost, institutional availability, convenience of use and patient factors such as the presence of severe reactive airway disease.

In addition to the valuable functional data obtained from stress CMR, CMR also provides highly accurate and reproducible structural information. CMR the gold standard for the assessment of ventricular chamber dimensions, volumes, and ejection fraction (EF) due to high temporal and spatial resolution and lack of ionizing radiation [5–7].

Published stress CMR protocols call for vasodilator induced first-pass perfusion imaging early in the study followed by cine acquisitions, typically 5–10 min after vasodilator administration, followed by late gadolinium enhancement sequences for infarct assessment although there is often variation in this practice [8, 9]. Adenosine and regadenoson are known to impact vital signs equivalently through an increase in heart rate and a reduction in blood pressure while the effects of regadenoson persist due to a longer half-life. Despite the changes in vital signs the effect on ventricular function is unknown and could have significant clinical implications [10]. In this analysis, we sought to define the duration and magnitude of change from baseline in biventricular volumes, dimensions, and EF in healthy subjects following administration of adenosine and regadenoson.

## Methods

### Patient population

A total of 25 healthy subjects between the ages of 18–45 years with normal electrocardiograms were prospectively enrolled to undergo a 6-min infusion of adenosine followed by regadenoson administration for assessment of changes in ventricular volumes and function by CMR. Patients were excluded if they reported a history of moderate to severe reactive airway disease, CAD, pulmonary hypertension, structural heart disease, theophylline use, contraindications to CMR, or were pregnant. All patient reported data was confirmed by study personnel via review of electronic medical records. Baseline demographic data to include age, gender, height, and weight were also collected. Study patients were instructed to refrain from caffeine consumption 48 h prior to CMR and presented the day of the study in a fasting state. All patients provided written informed consent and the study was approved by the San Antonio Military Medical Center institutional review board. No monetary compensation was provided for participation. DT and KS had full access to all the data in the study and takes responsibility for its integrity and the data analysis.

### CMR protocol and post-processing

All study participants underwent CMR on a 1.5 T CMR scanner (Siemens Magnetom Espree, Siemens Healthineers, Erlangen Germany [Tim 76 × 18]) with a 16 channel body coil (Invivo). Initial image sequences included localization and scout acquisitions. Baseline short axis (SAX) cine (steady state free procession) acquisitions (8 mm thickness, 2 mm skip) were acquired from the mid-atrial cavity level through the ventricular apex. This same SAX cine acquisitions were repeated immediately following conclusion of vasodilator stress administration (both adenosine and regadenoson) and at 5, 10, and 15 min after vasodilator administration. Intravenous gadolinium was not given. All CMR acquisition sequences were analyzed on a 3D workstation (QMass, Vital Images, Medis BV, Leiden, the Netherlands) by a cardiac imaging trained radiologist (MM) and Level III fellowship-trained CMR cardiologist (KS). Endocardial borders were defined for both the right ventricle (RV) and left ventricle (LV) using semi-automated software application with manual correction. Values were then indexed to body surface area (BSA) to determine LV end-diastolic volume (LVEDVi), LV end-systolic volume (LVESVi), LV ejection fraction (LVEF), RVEDVi, RVESVi, and RVEF.

### Stress protocol

**Fig. 1:**
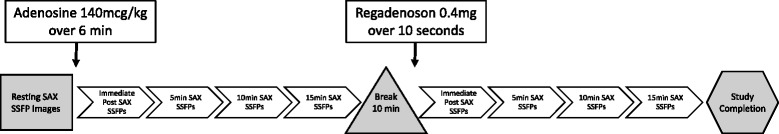
Schematic of the study design. SAX = short axis projection; SSFPs = steady-state free precession sequences. For the purposes of analysis, hemodynamic, volumetric, and ejection fraction results obtained at the 15 min point following adenosine administration were used as the baseline values for regadenoson administration

The stress protocol is outlined in Fig. 1. Abstinence from caffeine was verified orally on the day of the procedure. Following baseline SAX cine acquisitions, study participants received an infusion of adenosine (140mcg/kg/min IV for 6 min). As above, SAX cine stacks were obtained immediately following vasodilator administration and at 5 min, 10 min, and 15 min. Following the completion of the 15 min adenosine scan an additional washout period of 10 min was taken prior to administration of intravenous regadenoson (0.4 mg over 10 s) followed by the same SAX image acquisitions. Aminophylline was not given following regadenoson in accordance with published Society for Cardiovascular Magnetic Resonance (SCMR) protocols [9]. Blood pressure (BP), heart rate, and pulse oximetry (SpO2) were obtained at baseline, prior to each vasodilator administration and at the same time points as repeat image acquisition (immediately following vasodilator administration, 5 min, 10 min, and 15 min). For the purposes of assessing temporal changes following regadenoson administration, the volumetric and hemodynamic data acquired at the 15 min post-adenosine infusion mark were used as the baseline data for the regadenoson acquisitions. Following completion of image acquisition, study participants were surveyed regarding any side effects experienced with adenosine and regadenoson infusion and vasodilator preference.

### Statistical analysis

Parametrically distributed tabular data is presented as mean ± standard deviation (SD). A two-way repeated measures ANOVA was employed to assess differences between adenosine and regadenoson challenges on heart rate, BP and biventricular volumetric measurements and ventricular dimensions. *P*-values ≤0.05 were considered significant. Bonferroni post-hoc test was used to assess differences between adenosine and regadenoson at similar time points and the changes within treatments over time compared to baseline. Non-parametric analysis comparing baseline measurements to within treatment changes over time (0, 5, 10 and 15 min) was performed utilizing the Friedman repeated measures ANOVA with the Dunnett’s post-hoc test. Chi Square and Fisher’s Exact tests were employed to compare nominal data. Statistical analysis was performed utilizing SigmaPlot V12.5 (Systat Software, Inc. San Jose, California, USA).

## Results

### Study population

The study population consisted of 17 males and 8 females ranging in age from 28 to 42 years (mean 34 ± 5 years) with a mean body mass index (BMI) of 25.6 ± 4.1 kg/m2 (Table 1). No subjects had a history of theophylline use or caffeine consumption within 48 h. The baseline heart rate, BP and SpO2 were 64 ± 8 bpm, 136 ± 16/69 ± 12 mmHg, and 98 ± 1%, respectively. Eight subjects (32%) had initial baseline heart rates <60 bpm.

**Table 1:** Baseline Demographics

Study subjects (n)	25
Male, n (%)	17 (68%)
Age (years)	34 ± 5
BMI (kg/m2)	26 ± 4

Baseline patient demographics. Median values with standard deviation are shown for all continuous variables. BMI = body mass index

### Hemodynamic changes

**Fig. 2:**
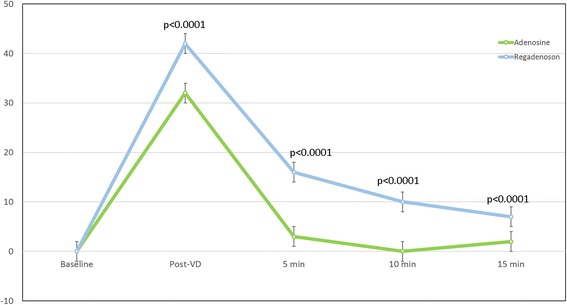
Heart rate changes following vasodilator administration: Temporal changes in heart rate from baseline following administration of adenosine (green line) and regadenoson (blue line). *P*-values shown represent differences in the magnitude of heart rate change at each time point between adenosine and regadenoson. The Post-vasodilator (VD) time point represents heart rates acquired immediately following vasodilator administration. Heart rate returned to baseline following adenosine infusion by the 10 min mark (*p* = 0.9397) whereas heart rate following regadenoson remained elevated compared with baseline at the 15 min mark (*p* = 0.0015)

Table 2 shows heart rate and BP changes with both adenosine and regadenoson. Administration of both vasodilatory agents resulted in an increase from baseline to peak heart rate immediately (Fig. 2) following vasodilator administration (adenosine: 64 ± 8 to 96 ± 13 bpm vs regadenoson: 65 ± 13 to 107 ± 10 bpm, *p* < 0.0001 for both). Conversely, heart rate returned to baseline by the 5 min mark following adenosine administration (*p* = 0.11) whereas a persistent, significant elevation in heart rate was observed in patients following 15 min after regadenoson administration (p < 0.0001). No significant durable effects were observed in systolic BP, diastolic BP, or Sp02 with all values returning to baseline by the 15 min mark following both regadenoson and adenosine administration.

**Table 2:** Vasodilator Induced Hemodynamic Changes

	Baseline	Immediate Post-VD	5 min	10 min	15 min	Change from Baseline at 15 min *p*-value
HR, bpm						
Adenosine	64 ± 8	96 ± 13	67 ± 8	64 ± 7	65 ± 13	0.4165
Regadenoson	65 ± 13	107 ± 10	81 ± 9	75 ± 7	72 ± 8	0.0015
Between agent p-value	0.4165	<0.0001	<0.0001	<0.0001	<0.0001	
SBP, mm Hg						
Adenosine	136 ± 16	131 ± 22	133 ± 18	134 ± 15	131 ± 24	0.1064
Regadenoson	131 ± 24	126 ± 17	126 ± 19	126 ± 19	129 ± 20	0.5998
Between agent p-value	0.3770	0.0780	0.7089	0.6514	0.8289	
DBP, mm Hg						
Adenosine	69 ± 12	69 ± 13	64 ± 10	65 ± 10	67 ± 11	0.3770
Regadenoson	67 ± 11	65 ± 12	63 ± 11	64 ± 10	67 ± 12	0.8289
Between agent p-value	0.3770	0.0780	0.7089	0.6514	0.8289	
Pulse oximetry, %						
Adenosine	98 ± 1	98 ± 2	98 ± 2	98 ± 2	98 ± 3	0.2204
Regadenoson	98 ± 3	98 ± 3	98 ± 1	98 ± 2	98 ± 1	0.1503
Between agent p-value	0.2204	0.1299	0.7449	0.7449	0.1503	
LVEF, %						
Adenosine	63.0 ± 4.6	69.8 ± 6.8	66.6 ± 5.6	63.3 ± 5.4	63.0 ± 6.9	0.9623
Regadenoson	63.0 ± 6.9	70.3 ± 5.0	69.8 ± 5.3	66.9 ± 5.9	67.2 ± 4.9	0.0015
Between agent p-value	0.9623	0.6842	0.0146	0.0068	0.0015	
LVEDVi, mL/m2						
Adenosine	71.2 ± 15.6	72.9 ± 15.4	70.2 ± 17.9	67.3 ± 16.0	66.3 ± 17.3	0.0023
Regadenoson	66.3 ± 17.3	64.5 ± 13.8	63.0 ± 14.4	62.9 ± 14.5	62.2 ± 14.9	0.0103
Between agent p-value	0.0023	<0.0001	<0.0001	0.0063	0.0103	
LVESVi, mL/m2						
Adenosine	26.4 ± 7.0	22.0 ± 6.3	23.7 ± 8.2	24.3 ± 7.9	25.6 ± 9.7	0.5226
Regadenoson	25.6 ± 9.7	19.1 ± 4.9	19.1 ± 6.1	21.1 ± 6.3	20.3 ± 5.5	<0.0001
Between agent p-value	0.5226	0.0136	0.0001	0.0074	<0.0001	
LVSVi, mL/m2						
Adenosine	44.8 ± 10.1	51.0 ± 11.8	46.6 ± 11.4	43.0 ± 10.4	41.9 ± 12.4	0.0584
Regadenoson	41.9 ± 12.4	45.4 ± 10.5	43.9 ± 9.9	41.8 ± 9.9	41.9 ± 10.9	0.9853
Between agent p-value	0.0584	0.0003	0.0857	0.4335	0.9853	
LV mass index, g/m2						
Adenosine	51.3 ± 9.1	56.6 ± 9.4	53.4 ± 11.0	52.5 ± 10.0	52.4 ± 11.3	0.4411
Regadenoson	52.4 ± 11.3	54.6 ± 10.8	53.5 ± 11.3	53.3 ± 11.7	53.5 ± 10.8	0.3937
Between agent p-value	0.4411	0.1538	0.9063	0.5165	0.3937	
RVEF, %						
Adenosine	57.0 ± 7.5	61.7 ± 6.9	59.1 ± 6.1	57.1 ± 7.3	58.5 ± 8.2	0.2905
Regadenoson	58.5 ± 8.2	62.4 ± 6.3	63.7 ± 7.1	61.9 ± 6.0	61.9 ± 7.5	0.0159
Between agent p-value	0.2905	0.6337	0.0014	0.0008	0.0159	
RVEDVi, mL/m2						
Adenosine	74.6 ± 16.7	78.3 ± 19.0	75.7 ± 18.7	72.4 ± 15.3	69.9 ± 17.5	0.0104
Regadenoson	69.9 ± 17.5	70.9 ± 17.1	66.4 ± 16.1	65.0 ± 14.5	66.0 ± 15.1	0.0357
Between agent p-value	0.0104	<0.0001	<0.0001	<0.0001	0.0357	
RVESVi, mL/m2						
Adenosine	32.1 ± 9.4	30.2 ± 9.4	30.8 ± 8.3	31.0 ± 8.1	28.7 ± 8.0	0.0024
Regadenoson	28.7 ± 8.0	26.6 ± 7.3	24.1 ± 7.8	24.6 ± 7.2	24.9 ± 6.7	0.0009
Between agent p-value	0.0024	0.0015	<0.0001	<0.0001	0.0009	
RVSVi, mL/m2						
Adenosine	42.5 ± 10.8	48.1 ± 12.1	44.9 ± 12.8	41.5 ± 10.7	41.2 ± 12.9	0.4339
Regadenoson	41.2 ± 12.9	44.3 ± 12.1	42.3 ± 10.9	40.0 ± 9.6	41.1 ± 11.5	0.9507
Between agent p-value	0.4339	0.0240	0.1175	0.3810	0.9507	

Hemodynamic, ejection fraction (EF), and ventricular volumetric changes following both adenosine and regadenoson. The Post-vasodilator time point represents data acquired immediately following vasodilator (VD) administration. All values are shown as mean ± standard deviation (SD). VD = vasodilator; HR = heart rate; SBP = systolic blood pressure; DBP = diastolic blood pressure; LVEF = left ventricular ejection fraction; LVEDVi = left ventricular end-diastolic volume indexed; LVESVi = left ventricular end-systolic volume indexed; LVSVi = left ventricular stroke volume indexed; LV mass = left ventricular end-diastolic mass indexed; RVEF = right ventricular ejection fraction; RVEDVi = right ventricular end-diastolic volume indexed; RVESVi = right ventricular end-systolic volume indexed; RVSVi = right ventricular stroke volume indexed

### Ejection fraction

**Fig. 3:**
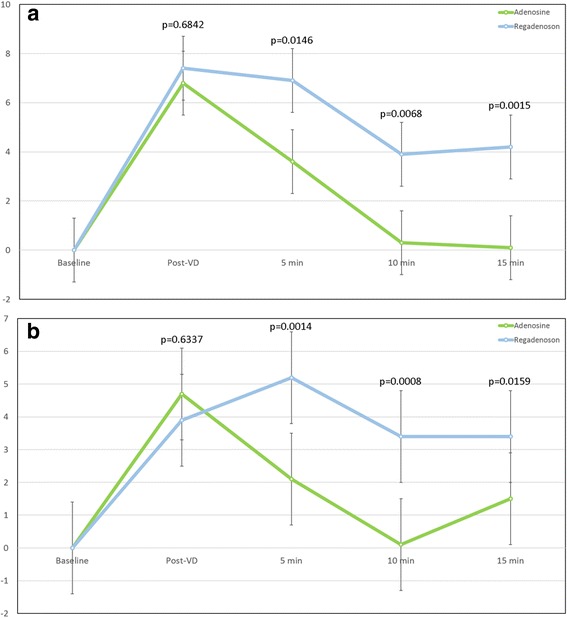
Changes in ejection fraction following vasodilator administration: Observed changes in left ventricular (LV) ejection fraction (LVEF) from baseline values following administration of adenosine (dashed line) and regadenoson (solid line) are shown in (**a**) and changes in right ventricular (RV) EF from baseline values are shown in (**b**). The Post-vasodilator time point represents short-axis cine data sets acquired immediately following vasodilator (VD) administration. *P*-values shown represent differences in the magnitude of EF change at each time point between adenosine and regadenoson. LVEF returned to baseline following adenosine infusion by the 10 min mark (*p* = 0.84) whereas LVEF following regadenoson remained elevated compared with baseline at the 15 min mark (p = 0.0015). Regarding RVEF (**b**), maximal impact of regadenoson occurred later than adenosine with adenosine returning to baseline by 15 min (*p* = 0.29). RVEF continued to be elevated at the 15 min mark following regadenoson administration (*p* = 0.016)

Administration of both adenosine and regadenoson resulted in an increase in left ventricular ejection fraction (LVEF) compared with baseline (Table 2). There was no significant difference in the peak increase in LVEF (Fig. 3a), which was observed immediately following administration of both vasodilators (*p* = 0.68), however LVEF following adenosine returned to baseline by the 10 min mark (baseline: 63.0 ± 4.6 vs 63.3 ± 5.4, *p* = 0.84). Conversely, LVEF following regadenoson administration remained increased when compared to baseline at the 15 min mark (baseline: 63.0 ± 6.9 vs 67.2 ± 4.9, *p* = 0.0015). This represents a mean increase in LVEF of 4.2 ± 1.3% baseline following regadenoson administration at the end of the sampling period (15 min mark).

Similar to LVEF, an initial increase was also observed in right ventricular ejection fraction (RVEF) following administration of both adenosine and regadenoson (Fig. 3b). Additionally, RVEF returned to baseline by the 5 min mark following adenosine administration (baseline: 57.0 ± 7.5 vs 59.1 ± 6.1, *p* = 0.13) while RVEF remained increased, though to a lesser degree when compared to effects on LVEF, following regadenoson administration out to 15 min (baseline: 58.5 ± 8.2 vs. 61.9 ± 7.5, *p* = 0.016).

### Ventricular volumes

**Fig. 4:**
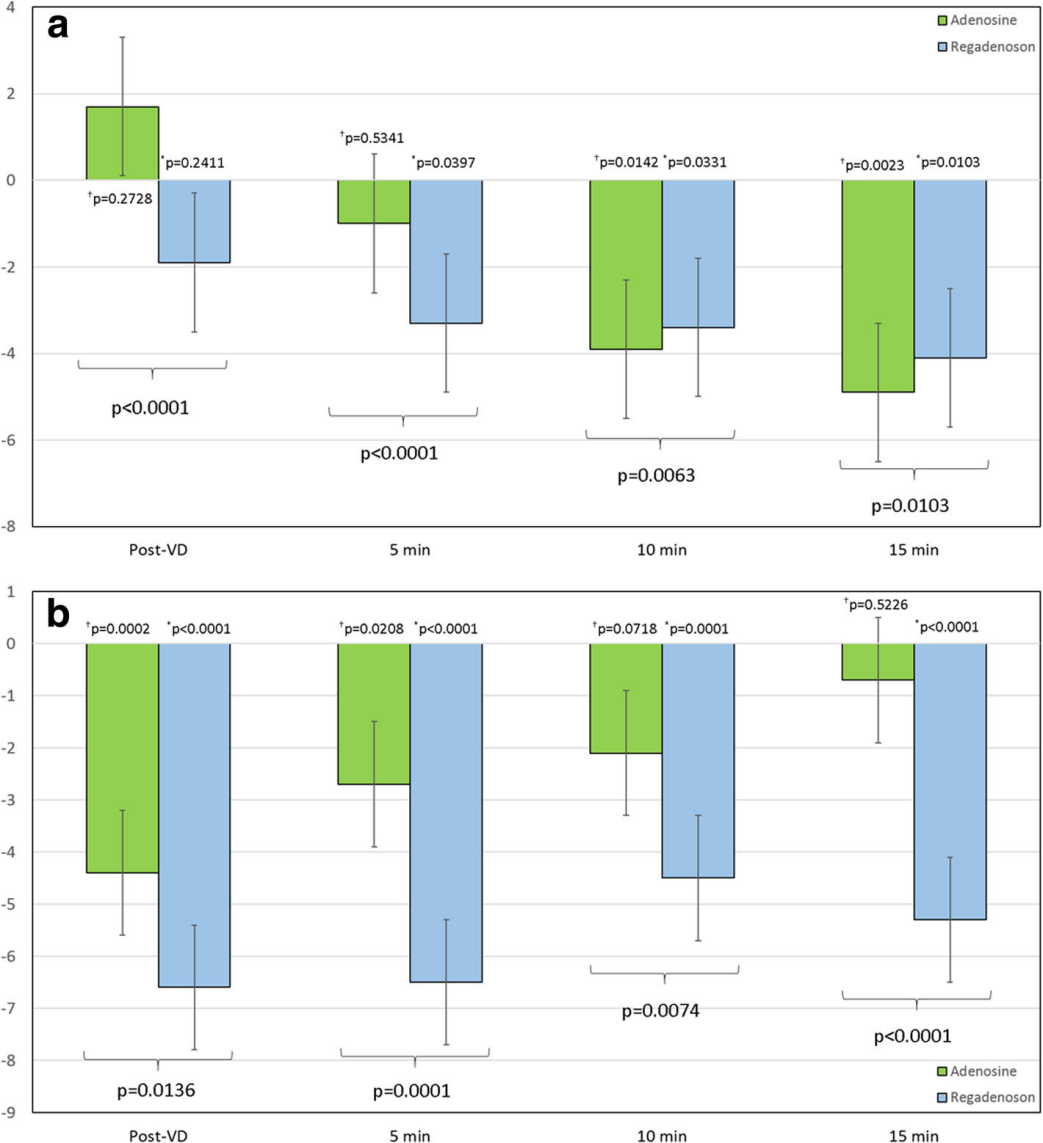
Temporal changes in left ventricular (LV) volumes from baseline: Observed changes in left ventricular end-diastolic volume index (LVEDVi) from baseline values following administration of adenosine (green bar) and regadenoson (blue bar) are shown in (**a**) and left ventricular end-systolic volume index (LVESVi) from baseline values are shown in (**b**). The Post-vasodilator time point represents data sets acquired immediately following vasodilator (VD) administration. P-values adjacent to the zero gridline represent changes from baseline at each time point following administration of regadenoson (*) and adenosine (†). P-values shown at the bottom of the figure represent differences in the magnitude of change in LVEDVi (**a**) and LVESVi (**b**) at each time point between adenosine and regadenoson

Adenosine and regadenoson were observed to have significant effects on LV end-diastolic volume index (EDVi) (Fig. 4a). LVEDVi dropped significantly when compared to baseline starting at the 10 min mark before reaching a nadir at the 15 min time point (net reduction in LVEDVi 4.9 ± 1.6 mL/m2, *p* = 0.002). LVEDVi trended downward following regadenoson administration, as well, reaching a nadir at the 15 min time point (net reduction in LVEDVi 4.1 ± 1.6 mL/m2, *p* = 0.010). When comparing the magnitude of LVEDVi reduction following both vasodilator agents, a greater reduction was observed following adenosine administration by the end of the protocol (p = 0.010).

An immediate, significant reduction in LV end-systolic volume index (ESVi) (Fig. 4b) was observed following administration of both adenosine and regadenoson (*p* = 0.0002 and *p* < 0.0001 for difference from baseline, respectively). Following adenosine infusion, however, LVESVi returned to baseline by the 10 min mark (baseline: 26.4 ± 7.0 mL/m2 vs 24.3 ± 7.9 mL/m2, *p* = 0.072) and remained as such through the 15 min period (*p* = 0.52). At termination of post-adenosine monitoring, the net decrease from baseline in LVESVi was 0.7 ± 1.2 mL/m2. Conversely, LVESVi remained decreased throughout the 15 min following administration of regadenoson (baseline: 25.6 ± 9.7 mL/m2 vs 20.3 ± 5.5 mL/m2, p < 0.0001). This translates to a 5.3 ± 4.2 mL/m2 reduction from baseline in LVESVi following regadenoson administration.

When combining these effects by looking at LV stroke volume indexed for body surface area (LVSVi), LVSVi following regadenoson returned to baseline levels within 5 min (*p* = 0.19 for net difference from baseline) of administration and remained as such through the 15 min observation period (*p* = 0.99 for net difference from baseline). This is explained by a fairly similar net reduction in both LVEDVi and LVESVi following regadenoson. Thus, with no change in LVSVi and a significant reduction in LVEDVi, a significant increase in LVEF would be expected and was observed. Conversely, LVSVi following adenosine infusion was trending toward significance (*p* = 0.058) with a net reduction of 2.9 ± 3.1 mL/m2 at the end of the observation period (15 min mark). This is explained by a more significant reduction in LVEDVi when compared to LVESVi following adenosine. Thus, LVSVi would be reduced to a similar magnitude to LVEDVi resulting in a negligible change in LVEF.

For completeness, temporal changes in RV volumes following adenosine and regadenoson administration are listed in Table 2. In general, the effects on RVEDVi and RVESVi following adenosine and regadenoson roughly mirror those seen in the LV volumes. Of note, LV mass, when indexed to BSA, predictably did not change significantly when assessed at all time points following administration of both adenosine and regadenoson.

### Side effects

**Fig. 5:**
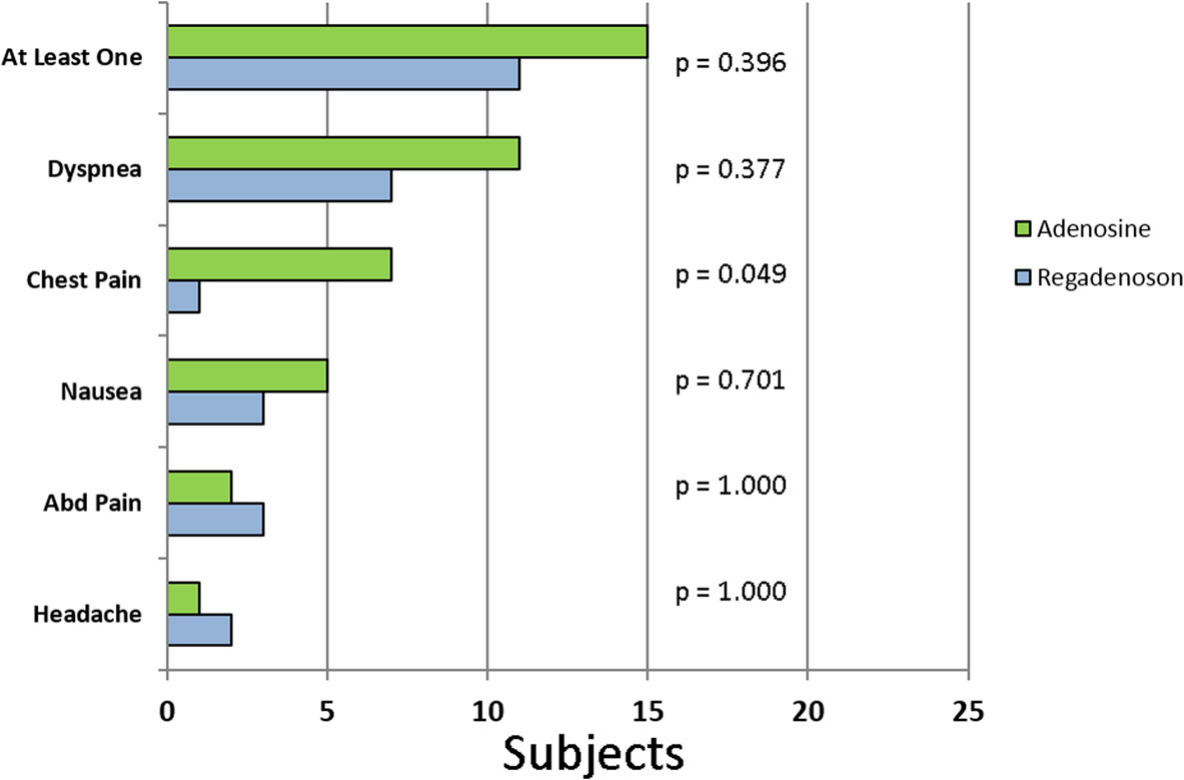
Patient symptoms following vasodilator administration: Patient reported symptoms experienced with administration of adenosine and regadenoson

Fifteen of the 25 subjects (60%) reported one or more side effects (Fig. 5) with adenosine compared and eleven (44%) with regadenoson (*p* = 0.40). Following administration of adenosine, 42% of the subjects reported dyspnea, 19% nausea, 4% a headache, 8% abdominal pain, and 27% chest pain. Following regadenoson, 44% of the subjects had dyspnea, 19% nausea, 12% a headache, 19% abdominal pain, and 6% chest pain. Only the incidence of chest pain differed significantly between adenosine and regadenoson (*p* < 0.049). When the total number of reported side effects were compared, a significant reduction in total side effects was noted with regadenoson (*p* = 0.015). Additionally, patient surveys revealed that subjects preferred >5:1 (*p* = 0.001) regadenoson over adenosine with respect to tolerability of side effects.

## Discussion

Our prospective, non-randomized crossover analysis of the cardiovascular impact of vasodilator agents on 25 healthy subjects is the first to report drug specific variations in cardiac physiology which ultimately could impact CMR quantitative results and clinical decision making. Current accepted stress CMR protocols allow for cardiac functional sequences to be performed either before or after vasodilator perfusion [9]. We found that performing these functional sequences after regadenoson resulted in a persistent mean increase in LVEF of 6.8%, 3.9%, and 4.3% at 5, 10, and 15 min post-injection. Conversely, following adenosine there was a mean LVEF increase of 3.3% at 5 min and minimal impact at 10 and 15 min post infusion. This effect on LVEF is explained largely by preserved stroke volume and a reduction in LVEDVi following regadenoson, but a reduction of stroke volume and LVEDVi following adenosine.

In most stress laboratories, stress CMR is performed utilizing either adenosine or regadenoson. Adenosine is a vasodilatory agent with an ultrashort half-life (<10 s) that is produced naturally in the body and nonselectively stimulates all known adenosine receptors (A1, A2A, A2B, and A3). Administration of intravenous or intracoronary adenosine results in super-physiologic augmentation of coronary blood flow (up to 500% above baseline). Regadenoson is a pyrazole derivative of adenosine and is significantly more selective for the adenosine A2A receptor found predominantly in the coronary vasculature. Administration of regadenoson results in similar levels of coronary hyperemia when compared to adenosine [3]. While the time to peak hyperemic effect is very similar to adenosine, metabolism in the plasma is slower (as long as 30 min) as it is not a substrate for adenosine deaminase or cell membrane nucleoside transporter [11]. Some CMR centers routinely use intravenous aminophylline to nonselectively block A1 and A2 adenosine receptors following regadenoson stress perfusion sequences in order to halt the physiologic effects of this agent. Dandekar et al. demonstrated that this was an effective method to quickly return myocardial blood flow to baseline measurements although the use of aminophylline as a reversal agent is considered off-label and not included in published CMR stress perfusion guidelines [9, 12]. Regardless of the vasodilator or protocol used, both vasodilator agents have been shown safe and effective when used with stress CMR [13, 14].

Although limited, other non-invasive stress modalities have evaluated the impact of vasodilator agents on cardiac function. Ohtaki et al. observed a significant reduction in LVEF 30 min following adenosine stress (baseline: 66 ± 8% vs stress: 63 ± 9%, *p* < 0.002) using single photon emission compuited tomography (SPECT), in addition to increases in LVEDV and LVESV [15]. In patients who underwent exercise stress in this analysis, no such changes to LVEF or LV volumes were observed [15]. To date, similar data has not been reported following regadenoson administration utilizing any modality, including SPECT, positron emission tomography (PET), or CMR. While small changes in EF may be of little consequence clinically amongst many patients, certain populations require maximum accuracy in LVEF measurement and this is a clear strength of CMR. For example, there are device guideline and Medicare reimbursement EF cutoff values for implantable cardiac defibrillator (ICD) implantation [16, 17]. Individuals with borderline cardiomyopathy undergoing CMR stress testing may have up to a 7% increase in EF simply due to vasodilator use and that increase could disqualify the implantation of a lifesaving device. Given our findings, modification of the stress CMR protocol to obtain ventricular function prior to vasodilator use (especially regadenoson) would be most appropriate when knowledge of ventricular function would have significant clinical implications. Aminophylline use immediately after regadenoson stress perfusion sequences may also be reasonable if ventricular functional sequences are performed afterwards [12]. In addition, using heart rate as a surrogate marker for resolution of vasodilator induced changes in cardiac physiology would be misleading as we were able to show persistent changes in cardiac function despite the heart rate returning to baseline values.

When compared with the package insert for adenosine, the incidence of chest pain (27% vs 40%) and headaches (4% vs 18%) was decreased in this analysis, respectively. This reduction was offset by an increased incidence of dyspnea (42% vs 28%) and nausea/abdominal pain (27% vs 13%) in study subjects when compared to the package insert, respectively [18]. Conversely, there was no discernable difference in the incidence of chest pain reported in this analysis when compared with the regadenoson package insert (6% vs 7%). The incidence of headache following regadenoson was reduced in our investigation (12% vs 26%) whereas dyspnea (44% vs 28%) and nausea/abdominal pain (38% vs 11%) were much more common in our analysis when compared to the package insert [19]. While interesting, these differences likely represent significant disparity between the sample sizes from which these side effect profiles are generated when compared with this analysis.

### Study limitations

Our study population was young and healthy therefore hemodynamic responses may not be similar to individuals with cardiovascular disease and/or myopathic processes. In addition, study subjects were instructed to refrain from caffeinated products 48 h prior to participation with oral verification at the time of the procedure and were not verified through serum analysis of caffeine levels. In an effort to limit significant alterations in plasma volume, subjects received two vasodilators during the same CMR session with a 25 min delay between injections of adenosine to the injection of regadenoson. Despite the fact that all easily detectable measures had returned to normal following adenosine administration (i.e. vital signs), not all of the ventricular volumes had returned to baseline, specifically LVEDVi, RVEDVi and RVESVi. This may impact the baseline comparison to post-regadenoson acquisitions. The use of the 15 min post-adenosine time point as the regadenoson baseline in lieu of performing a second, separate baseline data acquisition prior to administration of regadenoson may have resulted in some cross contamination with residual adenosine effects despite its short half-life of less than 10 s [18]. Reported side effects following regadenoson were increased compared to published data and may have been the result of consecutive vasodilator agent applications [19]. Some centers have incorporated the routine use of aminophylline following regadenoson stress to truncate hemodynamic effects as Dandekar et al. demonstrated rapid return to baseline of myocardial blood flow using this strategy [12]. While some CMR centers routinely use aminophylline as an abortive agent, we chose to follow the most current SCMR guidelines due to variations in this practice pattern. Our findings on the persistent augmented ejection fraction induced by regadenoson provides support for the use of an abortive agent if an accurate measurement of the EF is clinically impactful.

## Conclusions

Regadenoson and adenosine have a significant impact on ventricular volumes and EF following intravenous administration. The effects of regadenoson were demonstrated out to 15 min and adenosine out to 10 min. In patients undergoing vasodilator stress CMR where biventricular volumes and EF are critical components to the decision making process, ventricular functional sequences should be performed prior to the application of adenosine or regadenoson. Although not evaluated in this study, the augmented ventricular function induced by regadenoson may also support the use of aminophylline as a method to truncate these hemodynamic effects. Additionally, heart rate resolution can be misleading as it is not an effective surrogate marker for return of ventricular volumes and EF to baseline values following administration of a vasodilator stress agent.

## Abbreviations

BMI: Body mass index
BP: Blood pressure
CAD: Coronary artery disease
CMR: Cardiac magnetic resonance
EDVi: End diastolic volume index
EF: Ejection fraction
ESVi: End systolic volume index
ICD: Implanted cardiodefibrillator
PET: Positron emission tomography
Pox: Pulse oximetry
RV: Right ventricle/right ventricular
SAX: Short axis
SCMR: Society for Cardiovascular Magnetic Resonance
SD: Standard deviation
SPECT: Single-photon emission computed tomography
SSFPs: Steady state free precession sequence
SV: Stroke volume
T: Tesla
VD: Vasodilator

## References

[1] Paetsch I, Jahnke C, Wahl A, Gebker R, Neuss M, Fleck E, Nagel E (2004). Comparison of dobutamine stress magnetic resonance, adenosine stress magnetic resonance, and adenosine stress magnetic resonance perfusion. Circulation.

[2] Abbasi SA, Heydari B, Shah RV, Murthy VL, Zhang YY, Blankstein R, Steigner M, Jerosch-Herold M, Kwong RY (2014). Risk stratification by regadenoson stress magnetic resonance imaging in patients with known or suspected coronary artery disease. Am J Cardiol.

[3] Vasu S, Bandettini WP, Hsu LY, Kellman P, Leung S, Mancini C, Shanbhag SM, Wilson J, Booker OJ, Arai AE (2013). Regadenoson and adenosine are equivalent vasodilators and are superior than dipyridamole- a study of first pass quantitative perfusion cardiovascular magnetic resonance. Journal of cardiovascular magnetic resonance : official journal of the Society for Cardiovascular Magnetic Resonance.

[4] Hamon M, Fau G, Nee G, Ehtisham J, Morello R, Hamon M (2010). Meta-analysis of the diagnostic performance of stress perfusion cardiovascular magnetic resonance for detection of coronary artery disease. Journal of cardiovascular magnetic resonance : official journal of the Society for Cardiovascular Magnetic Resonance.

[5] Bellenger NG, Burgess MI, Ray SG, Lahiri A, Coats AJ, Cleland JG, Pennell DJ (2000). Comparison of left ventricular ejection fraction and volumes in heart failure by echocardiography, radionuclide ventriculography and cardiovascular magnetic resonance; are they interchangeable?. Eur Heart J.

[6] Shimada YJ, Shiota T (2011). A meta-analysis and investigation for the source of bias of left ventricular volumes and function by three-dimensional echocardiography in comparison with magnetic resonance imaging. Am J Cardiol.

[7] Rigolli M, Anandabaskaran S, Christiansen JP, Whalley GA (2016). Bias associated with left ventricular quantification by multimodality imaging: a systematic review and meta-analysis. Open Heart.

[8] Bettencourt N, Ferreira N, Chiribiri A, Schuster A, Sampaio F, Santos L, Melica B, Rodrigues A, Braga P, Teixeira M (2013). Additive value of magnetic resonance coronary angiography in a comprehensive cardiac magnetic resonance stress-rest protocol for detection of functionally significant coronary artery disease: a pilot study. Circulation Cardiovascular imaging.

[9] Kramer CM, Barkhausen J, Flamm SD, Kim RJ, Nagel E (2013). Society for Cardiovascular Magnetic Resonance Board of trustees task force on standardized P: standardized cardiovascular magnetic resonance (CMR) protocols 2013 update. *Journal of cardiovascular magnetic resonance: official journal of the Society for Cardiovascular Magnetic*. Resonance.

[10] Mekkaoui C, Jadbabaie F, Dione DP, Meoli DF, Purushothaman K, Belardinelli L, Sinusas AJ (2009). Effects of adenosine and a selective A2A adenosine receptor agonist on hemodynamic and thallium-201 and technetium-99m-sestaMIBI biodistribution and kinetics. JACC Cardiovascular imaging.

[11] Lieu HD, Shryock JC, von Mering GO, Gordi T, Blackburn B, Olmsted AW, Belardinelli L, Kerensky RA (2007). Regadenoson, a selective A2A adenosine receptor agonist, causes dose-dependent increases in coronary blood flow velocity in humans. Journal of nuclear cardiology: official publication of the American Society of Nuclear Cardiology.

[12] Dandekar VK, Bauml MA, Ertel AW, Dickens C, Gonzalez R, Farzaneh-Far A (2014). Assessment of global myocardial perfusion reserve using cardiovascular magnetic resonance of coronary sinus flow at 3 tesla. *Journal of cardiovascular magnetic resonance: official journal of the Society for Cardiovascular Magnetic*. Resonance.

[13] Karamitsos TD, Arnold JR, Pegg TJ, Cheng AS, van Gaal WJ, Francis JM, Banning AP, Neubauer S, Selvanayagam JB (2009). Tolerance and safety of adenosine stress perfusion cardiovascular magnetic resonance imaging in patients with severe coronary artery disease. The international journal of cardiovascular imaging.

[14] Nguyen KL, Bandettini WP, Shanbhag S, Leung SW, Wilson JR, Arai AE (2014). Safety and tolerability of regadenoson CMR. European heart journal cardiovascular Imaging.

[15] Ohtaki Y, Chikamori T, Igarashi Y, Hida S, Tanaka H, Hatano T, Usui Y, Miyagi M, Yamashina A (2008). Differential effects comparing exercise and pharmacologic stress on left ventricular function using gated Tc-99m sestamibi SPECT. Ann Nucl Med.

[16] Medicare National Coverage Determination: Implantable Automatic Defibrillators. In: *100–3.* Edited by (CMS) CfMMS; 2005.

[17] Epstein AE, DiMarco JP, Ellenbogen KA, Estes NA 3rd, Freedman RA, Gettes LS, Gillinov AM, Gregoratos G, Hammill SC, Hayes DL *et al*: 2012 ACCF/AHA/HRS focused update incorporated into the ACCF/AHA/HRS 2008 guidelines for device-based therapy of cardiac rhythm abnormalities: a report of the American College of Cardiology Foundation/American Heart Association task force on practice guidelines and the Heart Rhythm Society. *Journal of the American College of Cardiology* 2013, 61(3):e6–75. 10.1016/j.jacc.2012.11.007 23265327

[18] Adenosine (Adenoscan) [Package insert]. In: *Astellas Pharma.* Northbrook, IL; 2014.

[19] Regadenoson (Lexiscan) [Package insert]. In: *Astellas Pharma.* Northbrook, IL; 2014.

